# A cross-sectional survey of first-aid kit equipment in a family in Sichuan, China

**DOI:** 10.1186/s12889-024-19376-y

**Published:** 2024-07-09

**Authors:** Dan Wen, Li Wan, Haiyan He, Qianmei Zhong, Qingli Jiang, Xiuru Yang, Dan Zhang, Yuqi Shen

**Affiliations:** 1 Intensive Care Unit, Mianyang Central Hospital, School of Medicine, University of Electronic Science and Technology of China, Mianyang, Sichuan Province China; 2 Department of Nursing, Mianyang Central Hospital, School of Medicine, University of Electronic Science and Technology of China, Mianyang, Sichuan Province China

**Keywords:** First aid kit, Health literacy, Community residents, Influencing factor

## Abstract

**Purpose:**

To examine residents’ first-aid kit preparation and its influencing factors.

**Design:**

Cross-sectional survey.

**Methods:**

A questionnaire survey was conducted among 449 permanent residents in Sichuan Province using convenience sampling. We examined participants’ demographic characteristics, self-efficacy, health literacy, and personality.

**Findings:**

Of the participants, 111 (24.7%) stocked a home first-aid kit. The most frequent supplies were disinfection supplies (91.9%), common medicines (86.5%), and dressing supplies (76.6%). Family per capita monthly income, medical expenses payment method, chronic diseases, general self-efficacy, and health literacy were influencing factors of family first-aid kit preparedness.

**Conclusion:**

A multilevel and interactive emergency literacy education system should be established to improve residents’ abilities to prevent emergencies.

**Supplementary Information:**

The online version contains supplementary material available at 10.1186/s12889-024-19376-y.

## Introduction

Globally, 45 million people are disabled and 5.8 million die each year due to sudden trauma [1], the fourth leading cause of death. Injury-related death and disability impose a massive burden on low- and middle-income countries, comprising nearly 90% of the global injury toll [2]. The home first-aid kit is a comprehensive first-aid kit for accidents, such as knife wounds or earthquakes. They guarantee the safety of residents and play an important role when family members are injured [3]. In recent years, living standards have improved, and health awareness has been promoted. Home first-aid kits have been receiving increasing attention. In particular, owing to the impact of the COVID-19 pandemic, people’s attention and demand for medical and protective products have increased rapidly.

According to cognitive theory, self-efficacy is the strongest factor affecting public emergency preparedness behavior and a successful sustainer of health promoting behavior. A study [4] has found that individuals’ emergency preparedness behaviors are related to emergency knowledge, attitude, and self-efficacy. Findings from a study investigating the relationship between personality traits and hoarding behaviors in patients with novel coronavirus pneumonia, has found that, in emergency situations, agreeableness, neuroticism, and openness tend to lead to emergency supplies hoarding [5]. Health literacy refers to the ability of individuals to obtain and understand basic health information and make correct decisions to promote their health. There is a positive correlation between health literacy and health behaviors, including preventive measures and emergency service utilization [6].

American public health agencies work to help people respond to disasters and emergencies. In 2003, the U.S. Federal Emergency Management Agency launched a national emergency preparedness campaign to encourage the public to prepare emergency supplies [7]. In Australia, approximately one-fifth of households have mobile phones, first-aid kits, batteries, and other first-aid items [8]. Facing frequent extreme weather events and natural disasters, in 2015, China established its Ministry of Emergency Management, and issued the Emergency Management Standards. These standards encouraged households to stock first-aid kits and masks and aimed to improve the public’s awareness of disaster prevention and mitigation, as well as their ability to rescue themselves and others [9].

According to cognitive theory, self-efficacy is a pivotal factor influencing public emergency preparedness and health-promoting actions. However, the specific determinants motivating or impeding families in Sichuan to prepare and utilize first-aid kits remain unclear. This study aims to bridge the knowledge gap by exploring the relationship between emergency preparedness behaviors, personality traits, health literacy, and household first-aid kit preparedness/utilization in Sichuan, China, thereby offering insights for optimizing family emergency preparedness and first-aid kit utilization.

## Materials and methods

### Participants

Families with reading comprehension and expression skills who had lived in a city in Sichuan Province, China, for more than six months within the previous 12 months were included in this study. Participants with mental disorders and those who provided incomplete information were excluded. After receiving a detailed explanation of the study, each participant provided verbal informed consent.

### Questionnaire

The questionnaire comprised three parts. The first was the sociodemographic characteristics of the research participants, including gender, age, education level, per capita monthly income of the family, whether family members suffer from chronic diseases, and how medical expenses are paid. The second part of our study comprehensively evaluated the availability and completeness of home first-aid kits among participants’ households. Initially, we investigated the possession of a home first-aid kit, gaining insights into the percentage of households that have prepared a kit for emergencies. Subsequently, for households that confirmed the presence of a kit, we assessed its completeness by inquiring about the specific types of items within. The definition of a home first-aid kit is a collection of essential, well-stocked medical supplies, including bandages, analgesics, and antiseptic cream, readily accessible for self-care in minor household injuries or illnesses. The third component was the standard scale, which included the New General Self-Efficacy Scale (NGSES), Short-Form Health Literacy Instrument (HLS-SF12), and the 10-item short version of the Big Five Inventory (BFI-10).

### The new general self-efficacy scale

The NGSES was used to evaluate residents’ self-efficacy [10]. There are 10 items in the scale. A 4-point Likert scale was used, with overall scores ranging from 10 to 40. Higher scores indicate a higher sense of self-efficacy. The Cronbach’s α coefficient of this scale was 0.87, and the retest reliability in this study was 0.87.

### The short-form health literacy instrument

The HLS-SF12 was used to assess residents’ health literacy [11]. The scale includes 12 items regarding three aspects: medical care, disease prevention, and health promotion. Each item is rated on a 4-point scale (1 = very hard, 2 = hard, 3 = easy, and 4 = very easy). The higher the score, the higher the health literacy level. The Cronbach’s α coefficient of the scale was 0.87, and the retest reliability in this study was 0.86.

### The 10-item short version of the big five inventory

The 10-item short version of the BFI-10 was used to evaluate residents’ personality characteristics [12]. The scale includes ten items and five dimensions: neuroticism, extraversion, openness, agreeableness, and conscientiousness. The scale uses a 5-point Likert scale ranging from 1 (strongly disagree) to 5 (strongly agree); the higher the score, the more significant the personality traits. The internal consistency coefficients of the five dimensions of the scale ranged from 0.443 to 0.708, and the retest reliability ranged from 0.819 to 0.901.

### Data collection

The study was conducted from March 1 to May 1, 2023, using convenient sampling methods. To obtain a representative sample, we initially applied cluster sampling to identify distinct clusters in the community, selected based on geographical criteria. Subsequently, within these clusters, we utilized convenient sampling to individually administer questionnaires to residents. Through the assistance of community workers, the researchers entered the community and distributed questionnaires to the residents individually. Data were collected using an online questionnaire platform called Wenjuanxing, the most popular survey software in China (https://www.wjx.cn/).

### Data analysis

Data were analyzed using SPSS (version 25.0; IBM, Chicago, IL, USA) with a significance threshold of *p* < 0.05. Measurement data conforming to normal distribution are described as mean ± standard deviation. Measurement data with non-normal distribution are described as the median and quartile. The count data are described and analyzed based on frequency and component ratios. Demographics, self-efficacy, health literacy, and personality characteristics were independent variables in this study. The availability of a home first-aid kit was the dependent variable. The chi-square test and rank-sum test were used for single-factor analysis. Multivariate binary stepwise logistic regression was used for the multivariate analysis.

### Ethical considerations

The study was conducted in accordance with the principles of the Declaration of Helsinki. The first page of the questionnaire introduced the purpose and content of the study. The respondents were asked if they agreed to participate in the study. The participants had to click the “Agree” button to enter the questionnaire filling interface. Only those who agreed to participate completed the questionnaire. The study procedure was approved by the Ethics Committee of the Mianyang Central Hospital (S202303110-01).

### Quality control

To ensure the reliability and validity of our study, we implemented rigorous quality control measures. Prior to the survey, we conducted two pre-investigations to identify and resolve questionnaire design issues. Expert consultation was sought twice to refine our methods and mitigate bias. During data collection, trained investigators administered questionnaires face-to-face to clarify doubts and ensure response accuracy. Post-collection, a double-check process verified logical consistency and data accuracy. If singular or outlier values were identified, the original questionnaire was retrieved and verified with the investigator. These measures strengthened the reliability of our findings.

## Results

### Basic information of respondents and preparation of home first-aid kit

A total of 458 questionnaires were collected, of which 449 were valid, with an effective recovery of 98.0%. A total of 111 households (24.7%) were equipped with first-aid kits. The chi-square test showed that there were statistically significant differences in first aid kit equipment among people with different education levels, per capita monthly family income, medical expenses payment methods, and whether they suffered from chronic diseases (*p* < 0.05, Table 1).

**Table 1:** Social characteristics and home first aid kit readiness of respondents (*n* = 449)

Characteristic	Number(%)	Number of households with a first aid kit(%)	Number of households without a first aid kit(%)	χ^2^	*P*
Total number	449(100.0%)	111(24.7%)	338(75.3%)		
Gender				1.437	0.231
Male	134(29.8%)	45(33.6%)	89(27.9%)		
Female	315(70.2%)	88(66.4%)	227(72.1%)		
Age				2.321	0.557
17–44 years	313(69.7%)	93(29.7%)	220(70.3%)		
45–59 years	126(28.1%)	37(29.4%)	89(70.6%		
60–74 years	9(2.0%)	2(22.2%)	7(77.8%)		
≥ 75 years	1(0.2%)	1(100.0%)	0(0.0%)		
Degree of education				60.221^*^	<0.001
Primary school	8(1.8%)	0(0.0%)	8(100.0%)		
Junior middle school	64(14.3%)	2(3.1%)	62(96.9%)		
High school	100(22.3%)	9(9.0%)	91(91.0%)		
Technical secondary school	86(19.2%)	37(43.0%)	49(57.0%)		
Undergraduate	135(30.1%)	38(28.1%)	97(71.9%)		
Postgraduate and above	56(12.5%)	25(44.6%)	31(55.4%)		
Marriage				1.262	0.547
Unmarried	107(23.8%)	23(21.5%)	84(78.5%)		
Married	317(70.6%)	83(26.2%)	234(73.8%)		
Divorce	25(5.6%)	5(20.0%)	20(80.0%)		
Monthly household income, RMB				54.446^*^	<0.001
≤ 3,000	124(27.6%)	6(4.8%)	118(95.2%)		
3,001 ~ 6,000	156(34.7%)	36(23.1%)	120(76.09%)		
6,001 ~ 9,000	106(23.6%)	35(33.0%)	71(67.0%)		
>9,000	63(14.0%)	34(54.0%)	29(46.0%)		
Way of bearing medical expenses				39.676^*^	<0.001
self-paying	140(31.2%)	11(7.9%)	129(92.1%)		
Basic medical insurance	271(60.4%)	95(35.1%)	176(64.9%)		
Commercial health insurance	38(8.5%)	5(13.2%)	33(86.8%)		
Whether a family member has a chronic disease				34.443^*^	<0.001
No	425(94.7%)	93(21.9%)	332(78.1%)		
Yes	24(5.3%)	18(75.0%)	6(25.0%)		

*P < 0.05

### Type of items stored by responders with a home first-aid kit

The most commonly stocked item was “sterilized items (such as iodine),” with 102 households (91.9%) having such items stocked. The least stocked items were “special drugs (such as quick-acting heart-saving pills and emergency angina medication),” accounting for 37 households (33.3%). The 40 respondents who chose “other items” filled in other items stored in their home first-aid kit, including masks, plasters, and eye drops (Table 2).

**Table 2:** Category of home first-aid kit (*n* = 111)

Category	Number of households	Percentage
Disinfection articles (e.g. iodine)	102	91.9%
Commonly used drugs (e.g. non-steroidal anti-inflammatory drugs, antidiarrheal drugs)	96	86.5%
Dressing supplies (e.g. gauze, bandage)	85	76.6%
Other articles	40	36.0%
Special drugs (such as quick-acting heart-saving pills, emergency relief of angina)	37	33.3%

### Self-efficacy, health literacy, and big five personality scores of respondents

The NGSES, HLS-SF12, and BFI-10 scores are shown in Table 3. The rank sum test showed that self-efficacy, health literacy, health promotion, Big Five personality traits, openness, and agreeableness had significant effects on whether a participant stocked a home first-aid kit (*p* < 0.05).

**Table 3:** Self-efficacy, health literacy, big five personality scores and home first-aid kit equipment

Project	Number of entries	With home first-aid kit	Without home first-aid kit	Z	*P*
Self-efficacy	10	25(20,30)	21(19,24)	-6.479^*^	<0.001
Health literacy	12	35(32,36)	32(29,35)	-5.124^*^	<0.001
Health care scale	4	11(10,12)	11(9,12)	-1.789	0.074
Disease prevention scale	4	11(9,12)	11(9,12)	-0.083	0.934
Health promotion scale	4	12(12,14)	11(9,12)	-10.914^*^	<0.001
Big five personality	10	26(22,29)	23(21,26)	-3.283^*^	0.001
Extroversion	2	4(4,5)	4(4,6)	-0.522	0.600
Nervousness	2	6(4,7)	5(4,7)	-0.850	0.395
Preciseness	2	4(4,5)	4(3,5)	-0.160	0.873
Openness	2	5(4,7)	5(4,6)	-3.395^*^	0.001
Agreeableness	2	4(4,6)	4(3,5)	-2.590^*^	0.010

*P < 0.05

### Factors affecting home first-aid kit preparation

Statistically significant variables in the univariate analysis of general information, self-efficacy, health literacy, and the Big Five personality traits were included in the binary logistic regression analysis. The assignment method for the independent variables is shown in Supplementary Material 1. The results show that per capita monthly household income, medical expenses payment method, chronic disease, general self-efficacy, and health literacy were factors influencing the availability of first-aid kits in family households. These differences were statistically significant (*p* < 0.05; Table 4).

**Table 4:** Logistic regression analysis of influencing factors of home first-aid kit

Factor	B	Standard error	Wald χ^2^	*P*	OR(95%CI)
Constant	12.217	2.359	26.824	<0.001	—
Monthly household income, RMB (take ≤ 3000 as reference)					
3001 ~ 6000	2.312	0.757	9.314	0.002	0.099(0.022–0.437)
6001 ~ 9000	3.014	0.789	14.582	<0.001	0.049(0.010–0.231)
≥ 9000	3.687	0.809	20.774	<0.001	0.025(0.005–0.122)
Degree of education (take primary school as reference)					
Junior middle school	0.161	1.325	0.015	0.903	1.175(0.088–15.769)
High school	0.263	1.293	0.041	0.839	1.301(0.103–16.405)
Technical secondary school	1.212	1.283	0.893	0.345	0.298(0.024–3.679)
Undergraduate	0.882	1.267	0.485	0.486	0.414(0.035–4.954)
Postgraduate and above	0.110	1.314	0.007	0.933	0.896(0.068–11.765)
Way of bearing medical expenses (take self-paying as reference)					
Basic medical insurance	1.960	0.450	18.940	<0.001	0.141(0.058–0.341)
Commercial health insurance	2.059	0.896	5.284	0.022	0.128(0.022–0.738)
Whether a family member has a chronic disease (refer to No)	3.414	0.826	17.076	<0.001	30.387(6.018-153.437)
Self-efficacy	0.164	0.031	28.568	<0.001	0.849(0.799–0.901)
Health literacy	0.151	0.038	15.840	<0.001	0.860(0.798–0.926)
Big five personality	0.040	0.032	1.518	0.218	0.961(0.902–1.024)

## Discussion

This study shows that 24.7% of included participants’ households in Sichuan Province were equipped with first-aid kits. Household per capita monthly income, medical expenses payment method, chronic disease, self-efficacy, and health literacy are factors that influence family first-aid kit preparedness. A survey of emergency preparedness knowledge, attitudes, and behaviors of community residents in Heilongjiang Province [6] has shown that less than 5% (133/2686) of the respondents prepared basic emergency supplies. A study on the emergency preparedness behaviors of Japanese residents has shown that only 11% of households stocked a home first-aid kit [13]. In China and many other regions abroad, people must consider the preparation of first-aid kits and other household emergency supplies.

In 2020, the Ministry of Emergency Management of China issued a list of recommended household emergency supplies. The emergency medicine list includes commonly used medicines (over-the-counter drugs such as anti-infection, anti-cold, and anti-diarrhea drugs), medical materials (wound dressings such as bandages, band-aids, and gauze), betadine, and cotton swabs (for wound treatment and disinfection). Among the participants who had prepared a home first-aid kit, the most stocked item was disinfection supplies (91.9%). Among the surveyed households, 86.5% had stocks of commonly administered drugs. This was mainly possibly related to the policy of epidemic containment and control in the early stages and an increase in residents’ health awareness. Residents reserve drugs, mainly anti-inflammatory, anti-diarrheal, and other daily treatment drugs. However, s drugs such as rescue pills, traditional Chinese medicine, angina pectoris, and other emergency medicines are limited. This may be related to factors, such as whether a family member has a chronic disease. In addition, masks have become an important tool for preventing the spread of respiratory viruses, and many home first-aid kits (36%) are equipped with masks. Government departments should strengthen publicity and training, improve community residents’ preparedness for emergencies, and increase the public’s awareness of first aid.

The per capita monthly household income, the medical expenses payment method, and the presence or absence of chronic diseases are factors that were found to influence the availability of first-aid kits in households. Respondents with higher per capita household income were more likely to have a home first-aid kit. Similarly, a previous study has found that monthly household income was a factor affecting the behavior of residents in preparing emergency supplies [3]. The higher the monthly household income, the more conscious residents were regarding protecting their lives and property. Respondents who had health insurance were more likely to have a home first-aid kit than those who paid for their own medical expenses. It has been found that differences in medical expenses payment methods reflect differences in the medical care level received [14]. Respondents without health insurance were likely to be financially disadvantaged and have relatively low incomes, therefore being less likely to have a home first-aid kit. Respondents with chronic diseases are more active and self-manage their health [15]. Those with proactive access to health-related information and who make appropriate health decisions are more likely to stock a home first-aid kit; therefore, the Chinese government should implement measures to develop effective medical insurance policies, increase compensation, and gradually expand the scope and proportion of medical insurance reimbursement. The treatment of chronic diseases in outpatient clinics should be improved to reduce the cost burden on residents’ families. Furthermore, basic medical insurance, serious disease insurance, and medical assistance services should be provided to low-income rural residents.

Self-efficacy is an individual’s confidence in their ability to complete a specific task. It is closely related to an individual’s diet, physical exercise, smoking habits, and other health behaviors and is an important factor in promoting health [16]. Studies have shown that when self-efficacy is high, responses to emergencies show more positive attitudes [17]; therefore, higher self-efficacy is a protective factor for the public against emergencies. In our study, self-efficacy was one of the factors associated with the preparation of a home first-aid kits. People with higher self-efficacy were more likely to have a home first-aid kit than those with lower self-efficacy. Residents with high self-efficacy can remain calm when facing problems, form positive beliefs and attitudes, and stimulate their behavior. Improving self-efficacy is helpful in improving the level of health literacy and promoting the adoption of healthy behaviors and lifestyles.

Health literacy refers to an individual’s ability to obtain, understand, evaluate, and use information to make decisions and take actions that affect health conditions [9]. Health literacy is an important mediating variable that affects health outcomes, health behaviors, and access to and the utilization of medical services [18]. In this study, we found that respondents who scored higher on the health promotion dimension were more likely to have a home first aid kit than those who scored lower. This may be because the higher the degree of health promotion of the respondents, the more likely they are influenced by social or environmental factors to take positive health actions. People are increasingly using the Internet to obtain health information. This includes diverse sources, such as health professionals, the media, and social organizations. While providing quality medical information, the Internet and social media also increase the possibility of obtaining inaccurate, misleading, or commercially motivated medical information [19]. Researchers [20] have reviewed online health information and found that online health information quality is a major problem. They recommended improving the quality and accessibility of online information systems to help people effectively navigate to reliable health information sources.

This study has several limitations. First, we used convenience sampling, which may have introduced a selection bias. Second, our sample size was small and cannot represent the level of Sichuan Province or the whole country. Finally, we used a self-report questionnaire, which did not objectively reflect the authenticity of the participants’ relevant behaviors.

## Conclusion

Less than a quarter of families in Sichuan Province have first-aid kits stocked at home. Household per capita monthly income, medical expenses payment method, chronic diseases, general self-efficacy, and health literacy are factors that influence the availability of first-aid kits in households. Educational efforts should establish multilevel emergency literacy training and comprehensive public education programs, while policy initiatives should target income improvement, health insurance expansion, and financial incentives for first-aid kit purchases. Furthermore, community-based initiatives, collaborating with local stakeholders, should promote emergency preparedness and first-aid kit ownership. It is imperative to improve the public’s self-efficacy and health literacy; draw attention to the importance of emergency supplies, such as home first-aid kits; and increase residents’ abilities to prevent medical emergencies. Ultimately, a strengthened regulatory framework with minimum standards for first-aid kit availability, particularly for households with vulnerable members, is essential to ensure universal access.

### Electronic supplementary material

Below is the link to the electronic supplementary material.

Supplementary Material 1

## Data Availability

The datasets used and/or analysed during the current study are available from the corresponding author on reasonable request.

## Abbreviations

NGSES: New General Self Efficacy Scale
HLS: SF12 Short-Form Health Literacy Instrument
BFI: 10 10-item short version of the Big Five Inventory
