# Supplementary material for: A cross-sectional survey of first-aid kit equipment in a family in Sichuan, China
Source: BMC Public Health. 2024 Jul 9;24:1829. doi: 10.1186/s12889-024-19376-y (PMC11234814; doi:10.1186/s12889-024-19376-y)
Supplement: Supplementary file 1 — Supplementary Material 1 [file 12889_2024_19376_MOESM1_ESM.docx]

Supplementary materials 1. Logistic regression analysis variable assignment

| Factor | Variable Assignment |
| --- | --- |
| Degree of education | 1= Primary school, 2= Junior middle school, 3= High school, 4= Technical secondary school,5= Undergraduate, 6= Postgraduate and above |
| Monthly household income | 1=≤3000, 2=3001~6000, 3=6001~9000, 4=＞9000 |
| Way of bearing medical expenses | 1=self-paying, 2= Basic medical insurance, 3= Commercial health insurance |
| Whether a family member has a chronic disease | 0=No, 1=Yes |
| Self-efficacy | Actual value |
| Health literacy | Actual value |
| Big five personality | Actual value |
